# Non-shivering Thermogenesis Signalling Regulation and Potential Therapeutic Applications of Brown Adipose Tissue

**DOI:** 10.7150/ijbs.60354

**Published:** 2021-07-13

**Authors:** Zhengyan Zhang, Di Yang, Junwei Xiang, Jingwen Zhou, Hua Cao, Qishi Che, Yan Bai, Jiao Guo, Zhengquan Su

**Affiliations:** 1 Guangdong Engineering Research Center of Natural Products and New Drugs, Guangdong Provincial University Engineering Technology Research Center of Natural Products and Drugs, Guangdong Pharmaceutical University, Guangzhou 510006, China; 2 Guangdong Metabolic Diseases Research Centre of Integrated Chinese and Western Medicine, Guangdong Pharmaceutical University, Guangzhou 510006, China; 3 Guangdong Cosmetics Engineering & Technology Research Center, School of Chemistry and Chemical Engneering, Guangdong Pharmaceutical University, Guangzhou 510006, China; 4 Guangzhou Rainhome Pharm & Tech Co., Ltd., Guangzhou 510663, China; 5 School of Public Health, Guangdong Pharmaceutical University, Guangzhou 510310, China

**Keywords:** Thermogenesis, Signalling Pathways, Obesity, Brown Adipose Tissue, Natural products

## Abstract

In mammals, thermogenic organs exist in the body that increase heat production and enhance energy regulation. Because brown adipose tissue (BAT) consumes energy and generates heat, increasing energy expenditure via BAT might be a potential strategy for new treatments for obesity and obesity-related diseases. Thermogenic differentiation affects normal adipose tissue generation, emphasizing the critical role that common transcriptional regulation factors might play in common characteristics and sources. An understanding of thermogenic differentiation and related factors could help in developing ways to improve obesity indirectly or directly through targeting of specific signalling pathways. Many studies have shown that the active components of various natural products promote thermogenesis through various signalling pathways. This article reviews recent major advances in this field, including those *in* the cyclic adenosine monophosphate-protein kinase A (cAMP-PKA), cyclic guanosine monophosphate-GMP-dependent protein kinase G (cGMP-AKT), AMP-activated protein kinase (AMPK), mammalian target of rapamycin (mTOR), transforming growth factor-β/bone morphogenic protein (TGF-β/BMP), transient receptor potential (TRP), Wnt, nuclear factor-κ-light-chain-enhancer of activated B cells (NF-κΒ), Notch and Hedgehog (Hh) signalling pathways in brown and brown-like adipose tissue. To provide effective information for future research on weight-loss nutraceuticals or drugs, this review also highlights the natural products and their active ingredients that have been reported in recent years to affect thermogenesis and thus contribute to weight loss via the above signalling pathways.

## Introduction

Obesity has increasingly become a burden on the human health system. Effective treatment of obesity greatly reduces the morbidity of other metabolic diseases 1. Obesity is the excess storage of metabolic energy. Energy homeostasis is closely related to the active function of adipose tissue. Adipose tissue in animals consists mainly of brown adipose tissue (BAT) and white adipose tissue (WAT). BAT includes classic BAT and recently discovered beige adipose tissue, which resists cold and obesity through adaptive heat production, known as non-shivering thermogenesis. Non-shivering thermogenesis is regulated by multiple signalling pathways. Thermogenic adipocytes, including brown adipocytes and beige adipocytes, have been shown to be prolific in human infants and adults 2. Increasingly, it is becoming clear that BAT and BAT-like tissues have significant effects on total body metabolism in humans.

Signalling pathway-mediated control of the adipocyte lineage has been studied extensively. Thermogenesis regulation has long been assumed to mainly occur through typical pathways, such as the cyclic adenosine monophosphate-protein kinase A (cAMP-PKA) signalling pathway in the sympathetic nervous system (SNS). AMP-activated protein kinase (AMPK) promotes brown adipocyte differentiation and thermogenesis. mammalian target of rapamycin (mTOR), which antagonizes AMPK, also regulates brown adipocyte differentiation and thermogenesis. Transient receptor potential (TRP) channels can affect BAT thermogenesis by mediating Ca^2+^ influx in different ways. Other signalling pathways, including the Wnt, nuclear factor-κ-light-chain-enhancer of activated B cells (NF-κΒ), Notch and Hedgehog (Hh) signalling pathway, can also influence BAT differentiation and thermogenesis.

To realize and fully utilize the potential of these cellular metabolic functions, the evolutionary history and different regulatory mechanisms of thermogenesis should be explored. This review illustrates the current findings on all the thermogenesis-related signalling pathways that have been studied in recent years. In this paper, the role of specific signalling pathways in adipose tissue function regulation and their molecular mechanism are reviewed. In addition, this review illustrates the potential therapeutic applications, mainly focusing on natural products and their active ingredients that might improve obesity by affecting related signalling pathways.

## UCP1-mediated thermogenesis and transcriptional regulators in BAT

UCP1 is considered the origin of adaptive thermogenesis in BAT. UCP1 lowers the proton gradient by uncoupling the respiratory chain and facilitates synthesis of cyclic adenosine monophosphate (cAMP) which promotes thermogenesis capacity in mitochondria. Therefore, activation of UCP1 is an important protective mechanism against obesity and related metabolic diseases.

When adipocytes are activated, the first step is the release of norepinephrine (NE) by the SNS. Subsequently, adrenergic receptors (mainly β1 and β3 subtypes) are activated, therefore, cAMP increases greatly and leads to activation of PKA. PKA induces lipolysis, and releases free fatty acids (FFAs) that stimulate UCP1 expression in the inner mitochondrial membrane. UCP1 enables the active transport of protons across the semipermeable membrane by the electron transport chain to decrease the proton concentration gradient, thereby releasing energy instead of through ADP phosphorylation. Activated UCP1 rapidly induces movement of protons back into the mitochondrial matrix, which in turn reduces hydrogen levels, activates respiratory pathways and oxidizes fatty acids (FAs). In this situation, the proton pathway is not used to phosphorylate ADP to generate heat 3.

Although UCP1 is a decisive factor associated with thermogenic activity, thermogenesis is also influenced by many other factors in BAT, such as oxidative phosphorylation, FA metabolism, lipolysis and mitochondrial biogenesis 4. Accordingly, the biological significance of improving UCP1 transcriptional levels in BAT is also determined by other elements. The synthesis of rodent UCP1 requires other hormones in addition to NE, such as triiodothyronine, leptin, insulin, glucocorticoid, and corticosterone 5.

Transcriptional cascades that control the process of brown and beige adipocyte development induce a number of regulators. Element-binding proteins (CEBPs) and peroxisome proliferator-activated receptor γ (PPARγ) are the major moderators that control the fate of brown adipocytes 6, and PPARγ coactivator-1α (PGC-1α) is the major thermogenesis* factor* in adipocytes 7. Additionally, PR domain zinc finger protein (PRDM16) regulates PPARγ, CEBPs and PGC-1α, and bilaterally determines brown adipocyte differentiation 8. A number of upstream regulators of PRDM16, such as EBF2 and EHMT1, positively induce browning of adipocytes 9, 10, and negative regulators, including TLE3 and miRNAs, lead to the opposite results 11. Forkhead box C2 (FoxC2) also induces browning of WAT in response to leptin-STAT3-PRDM16 signalling 12. In summary, many transcription factors in BAT, such as PGC-1α, PRDM16, PPARγ, CEBPs and FoxC2, control thermogenesis in BAT or beige fat *via* UCP1-mediated mechanisms. How synergy and negative feedback occur among these factors or whether the coexisting thermogenesis signalling pathways in vivo have causal relationships with these mechanisms is unknown, surely, these pathways must have common connections.

## Major advances in thermogenesis signalling pathways in BAT

In the past few decades, numerous studies have identified positive or negative regulators involved in the development of brown adipocytes 13. β-AR signalling is a dominant pathway involved in energy balance and thermogenesis that contributes to thermogenesis in BAT and browning of WAT. Furthermore, previously unobserved signalling pathways have recently been reported. Here, we review recent studies on signalling pathways that are responsible for controlling thermogenesis in BAT and beige fat.

### cAMP-PKA signalling pathway

The cAMP-PKA signalling pathway is the most classical pathway in thermogenesis and has been studied in depth. UCP1 activation and transcription in BAT are regulated by NE, which is released from the SNS. NE binds to β-AR through the p38 mitogen-activated protein kinase (p38 MAPK) signalling pathway to activate AC coupled to G proteins (i.e., guanine nucleotide-binding regulatory proteins, including GDPα, GDPβ, and GDPγ). This increases the concentration of intracellular cAMP, which is a secondary messenger in the cell. As a result, PKA is phosphorylated, followed by p38 MAPK activation. This activation leads to hormone-sensitive triglyceride lipase (HSL) phosphorylation, which ultimately promotes decomposition of triglycerides stored in lipid droplets into glycerol and FA and activates UCP1expression.

There are two classical pathways by which PKA regulates thermogenesis. In one pathway, UCP1 expression is upregulated by PKA in a p38 MAPK-dependent manner. PKA activates two important downstream substrates, PGC-1α and activating transcription factor-2 (ATF-2). On the one hand, activation of p38 MAPK enables phosphorylation and activation of ATF-2 via cAMP in response to CREB to promote PGC-1α and UCP1 transcription in BAT. In addition, activation of p38 MAPK phosphorylates PGC-1α and activates PGC-1α to induce UCP1 transcription by binding to PPARγ and the UCP1 promoter. In the p38 MAPK-independent process, CREB is phosphorylated by PKA and binds with cAMP to directly promote UCP1 and PGC-1α expression, promoting the occurrence of beige fat and enhancing thermogenesis 14.

In the other pathway, lipohydrolysis is promoted. FAs are both substrates and activators of thermogenesis in BAT. PKA can activate HSL and adipose triglyceride lipase (ATGL), promoting its lipolysis function, which increases the release of FFAs for mitochondrial utilization, thereby regulating BAT thermogenesis. Perilipin (PLIN) exists on the surface of lipid droplets, and can block the contact between lipid droplets and lipase, acting as a barrier to the lipid decomposition reaction. PKA phosphorylation of PLIN removes this barrier effect, allowing lipid droplets to fully contact ATGL and initiating lipohydrolysis 15.

In addition to the classical methods, many new methods by which cAMP-PKA regulates BAT thermogenesis have recently been found. Under endoplasmic reticulum stress, PKA phosphorylates inositol-requiring enzyme-1α (IRE-1) and IRE-1 subsequently activates X-box binding protein-1 (XBP-1) which has transcription factor activity and can upregulate UCP1 expression and increase BAT thermogenesis 16. Silent information regulator-1 (Sirt-1) is an important transcriptional regulator. PKA activates SIRT-1, which in turn activates PGC-1. PGC-1 upregulates the expression of thermogenesis-related genes and thereby increases thermogenesis 17. Adaptor protein containing the pleckstrin homology domain, phosphotyrosine binding domain and leucine zipper motif (APPL1), stimulated by cAMP, travels from the cytoplasm to the nucleus and interacts directly with histone deacetylase 3 (HDAC3) to mediate UCP1 expression in cultured brown fat cells 18. The microbiota has also been found to promote thermogenesis by activating cAMP-PKA signalling 19. A recent study confirmed that outer mitochondrial membrane-located AIDA is phosphorylated by PKA, translocates to the intermembrane space and activates UCP1 expression and thermogenesis 20.

Multiple factors can also negatively regulate thermogenesis by inhibiting the cAMP-PKA pathway. Insulin-AKT signalling is inhibited by phosphodiesterase (PDE), which is a classical pathway that negatively regulates lipohydrolysis. The cGMP-AMP (cGAMP) synthase-stimulator of interferon genes (cGAS-STING) pathway activated by mitochondrial stress inhibits PKA signal transduction by activating PDE, thus inhibiting BAT thermogenesis 21. RNA-binding protein quaking (QKI), induced by CREB, can reduce the stability, nuclear export, and translation of mRNAs encoding UCP1 and PGC1α, thereby restricting BAT energy expenditure 22.

**Figure 1:**
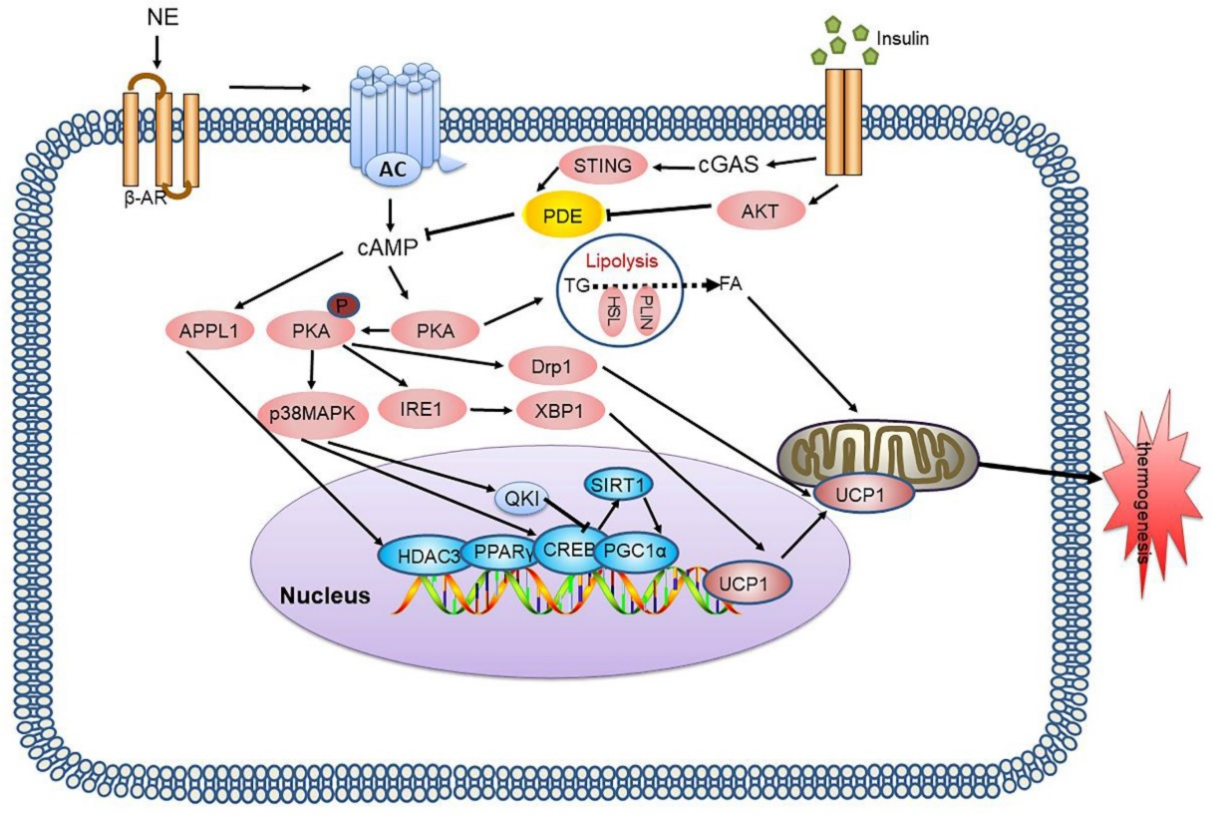
** cAMP-PKA signalling pathway and its signalling networks.** Sympathetic neurons release NE and activate β-AR on adipocytes, thereby activating AC which catalyses cAMP, resulting in PKA phosphorylation and activation of HSL and other components of the lipolysis pathway. PKA also activates the p38 MAPK pathway, which leads to increased UCP1 transcription and the expression of other prothermogenic genes. New regulatory factors affecting thermogenesis via the cAMP-PKA pathway are also continuously being discovered, including IRE-1, APPL1, and Drp1. The cGAS-STING pathway activated by mitochondrial stress inhibits PKA signal transduction by activating PDE, thus inhibiting BAT thermogenesis. NE: norepinephrine; β-AR: β-adrenergic receptor; AC: adenylyl cyclase; cAMP: cyclic adenosine monophosphate; PKA: protein kinase A; HSL: hormone-sensitive triglyceride lipase; p38 MAPK: p38 mitogen-activated protein kinase; UCP1: uncoupling protein 1; IRE-1: inositol-requiring enzyme-1α; APPL1: adaptor protein containing the pleckstrin homology domain, phosphotyrosine binding domain and leucine zipper motif; Drp1: dynamin-related protein 1; cGAS: cGMP-AMP (cGAMP) synthase; STING: stimulator of interferon genes; PDE: phosphodiesterase; BAT: brown adipose tissue

cAMP-PKA is the most intensively studied signalling pathway in BAT thermogenesis. It plays a key regulatory role in lipid metabolism and is an important target for treatment of lipid metabolism disorders and related diseases. Many regulatory factors based on this pathway are still being discovered, and it is hoped that understanding of this pathway can be continuously improved through future studies, to provide a research basis for treatment targeting this signalling pathway **(Figure 1)**.

### cGMP-AKT signalling

Apart from cAMP, cGMP is also an important second messenger. cGMP is produced by guanylyl cyclases (GCs) that are activated by nitric oxide (NO) or natriuretic peptides (NPs). cGMP activates GMP-dependent protein kinase G (PKG/AKT), and plays an important role in BAT thermogenesis 23. GCs can be divided into soluble and membrane bound proteins. Soluble GCs (sGCs) are heterodimers made up of α and β subunits, and are activated by NO. Nitric oxide synthases mediate endogenous NO production by converting L-arginine into citrulline, and NO, nitrate (NO^3-^) and nitrite (NO^2-^) are inert end products of NO metabolism 24, 25.

Many studies have demonstrated that the NO-cGMP-dependent pathway regulates mitochondrial biogenesis and energy balance. Linda focused on the sGC-dependent pathway, and found that an sGC pharmacological stimulator improves obesity and leads to positive metabolic changes 26. Becerril et al. showed that ablation of inducible NOS improved the energy balance in ob/ob mice by increasing thermogenesis 27. Gursimran suggested that nonburning (low-dose) UVR inhibits WAT generation and steatosis through skin release of NO 28. NO has been shown to directly prevent mitochondrial respiration by occupying the oxygenation site of cytochrome oxidase 29.

**Figure 2:**
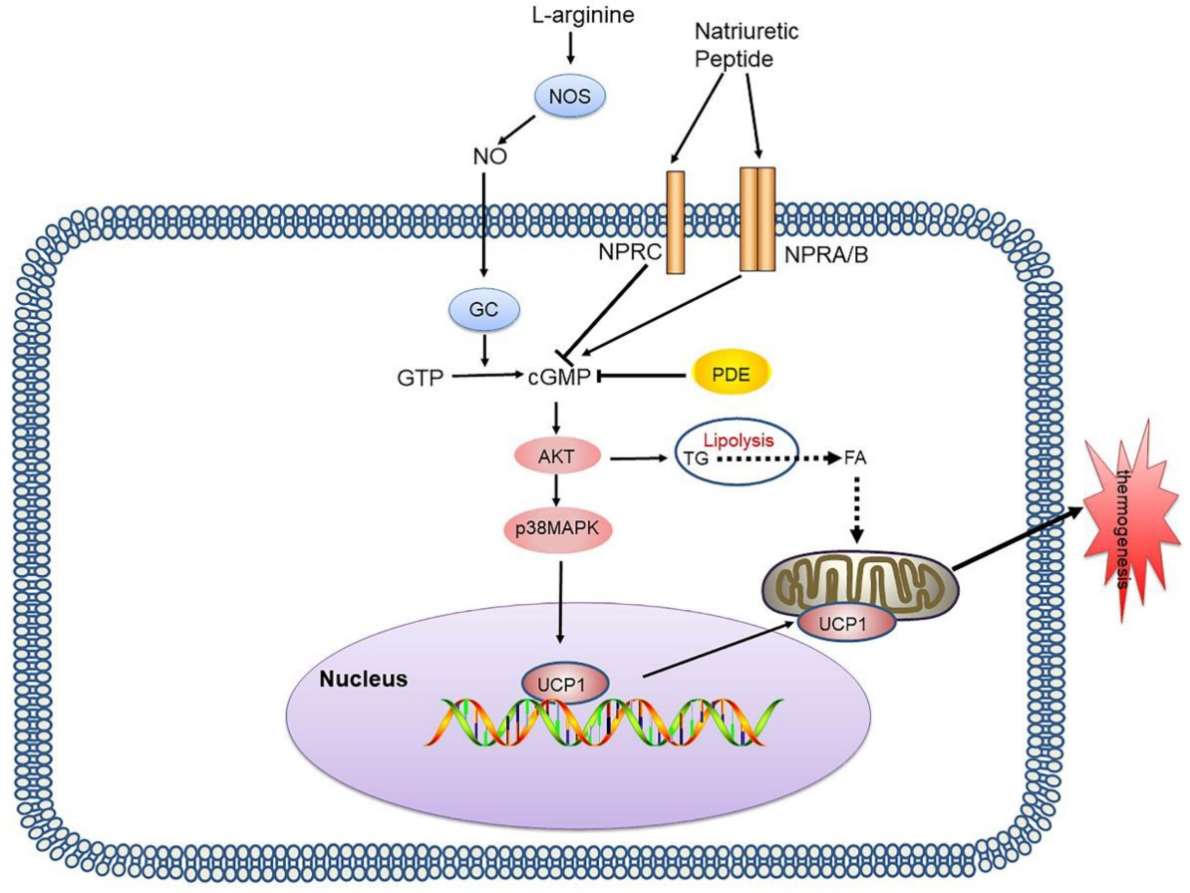
** cGMP-AKT signalling pathway and its signalling networks.** Nitric oxide synthases mediate endogenous NO production by converting L-arginine into citrulline. NO activates GCs in adipocytes, thereby increasing the intracellular cGMP concentration which results in AKT phosphorylation and activates lipolysis and UCP1 expression. NP binds to NPR and induces cGMP production, due to its GC activity. PDE also inhibits cGMP and inhibits thermogenesis. NO: nitric oxide; GCs: guanylyl cyclases; cGMP: cyclic guanosine monophosphate; AKT: protein kinase A; NP: natriuretic peptide; NPR: natriuretic peptide receptor

The NP system consists of three different ligand-receptor pairs, namely atrial natriuretic peptide (ANP), brain natriuretic peptide (BNP), and C-type natriuretic peptide (CNP), and the corresponding receptors (NPRs): NPRA, NPRB, and NPRC 30. NP binds to NPR and induces cGMP production, and due to their GC activity, NPRA and NPRB are also referred to as particulate GCs. The action of ANP and BNP is mediated by the receptor NPRA, while NPRC binds to ANP and BNP, removing them from circulation 31** (Figure 2)**.

Recent studies have shown that CNP activates the BAT thermogenesis program in mouse and human adipocytes via p38 MAPK 32, and that ANP directly increases mitochondrial uncoupling and thermogene expression in human WAT and BAT 33. Anja et al. studied the effect of an optimized designer natriuretic peptide (CD-NP) on adipose tissue in mice and found that WAT browning increased in mice treated with CD-NP for 10 days. However, long-term treatment with CD-NP led to weight gain, reduced glucose tolerance, reduced lipolytic activity, and cirrhosis 34. The effects of NPs vary with treatment duration, and the mechanism is unknown. cGMP-regulated pathways remain less studied, and many questions remain about the mechanisms that we now know about.

### AMPK signalling pathway

AMPK, a ubiquitously distributed serine/threonine protein kinase, is a crucial regulator involved in multiple metabolic pathways, is highly expressed in both the brain and BAT, and regulates thermogenesis. AMPK activation is determined by its heterotrimeric structure, comprising of a catalytic subunit (α1 and α2) and two regulatory subunits (β and γ) 35. AMPK has an important role in improving glucose uptake, FA oxidation, and mitochondrial biogenesis to treat obesity and other metabolic diseases 36. AMPK activation rewires metabolism to reduce ATP consumption and increase ATP production to favour energy balance 37.

In BAT, AMPK activation facilitates glucose and FA uptake, improves mitochondrial function and FA oxidation, increases non-shivering thermogenesis and inhibits fat and cholesterol synthesis in brown and beige adipocytes. Zhao, et al. showed that AMPK deficiency decreases progenitor cell density, inhibits brown adipocyte differentiation, and promotes fibrous cell differentiation 38. Wu et al. found that adipocyte AMPKα deficiency inhibits thermogenesis and energy consumption when stimulated by cold and β-AR, resulting in obesity and related metabolic disease 39. NE increases PGC-1α expression in the same manner as activation of AMPK signalling in the presence of AMPKβ1 40.

AMPK is also important for maintaining the normal function of BAT mitochondria. Mitochondrial Ca^2+^ plays an important role in regulating mitochondrial activity, and the mitochondrial calcium uniporter (MCU) is the most critical channel that mediates Ca^2+^ uptake. A recent study reported that the expression levels of MCU complex members were increased during obesity in mice and human adipose tissues 41. Gao, et al. found that capsaicin could activate the AMPK-SIRT3 positive-feedback loop to epigenetically inhibit MCU-dependent mitochondrial Ca^2+^ overload in brown adipocytes, thus maintaining the morphology and function of BAT against whitening stimuli 42.

Notably, different subunit combinations can potentially form functionally distinct complexes with distinct substrate specificities. However, as far as the current study is concerned, no studies in animal models have considered this question. Moreover, there are still many mechanisms through which AMPK affects BAT activity, such as creatine and calcium shuttles which are UCP1-independent thermogenesis pathways.

**Figure 3:**
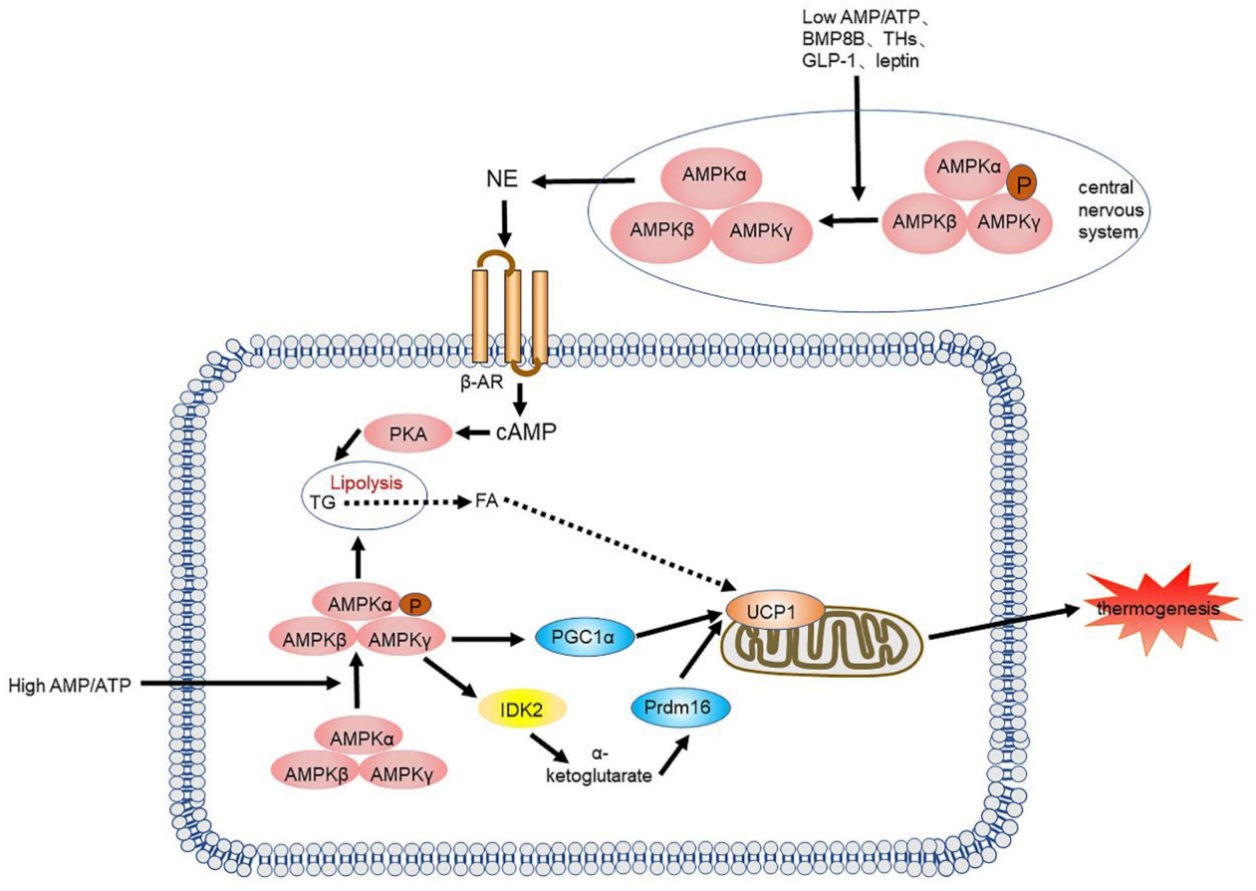
** AMPK signalling pathway and its signalling networks.** Low AMP/ATP ratios, BMP8B, THs, GLP-1 and leptin inhibit AMPK activity in the hypothalamus, leading to NE release, which leads to an increase in adipogenesis and thermogenesis through the cAMP-PKA signalling pathway. A high AMP/ATP ratio activates AMPK, resulting in increased UCP1 transcription and the expression of other thermogenic genes. AMPK also increases the activity of the TCA cycle enzyme IDH2, yielding α-KG, which leads to demethylation of Prdm16 and results in thermogenesis. BMP8B: bone morphogenetic protein 8B; THs: thyroid hormones; GLP-1: glucagon-like peptide-1; AMPK: AMP-activated protein kinase; NE: norepinephrine; TCA: tricarboxylic acid; IDH2: isocitrate dehydrogenase 2; α-KG: α-ketoglutarate; Prdm16: PR domain zinc finger protein16

AMPK is also essential in the central nervous system (CNS), especially in the hypothalamus. Recently, increasing evidence has suggested that numerous hormonal factors, such as leptin 43, 44, thyroid hormones 45, 46, and glucagon-like peptide-1(GLP-1) 47-49 control BAT differentiation and thermogenesis by suppressing hypothalamic AMPK activity. Collazo, et al. showed that inhibiting AMPK in the ventromedial nucleus of the hypothalamus can counter high fat diet (HFD)-induced obesity by activating BAT thermogenesis and subsequently energy consumption 50. Rosalía et al. recently demonstrated that carnitine palmitoyltransferase I(CPT1C) may be a downstream factor of AMPK that regulates hypothalamic thermogenesis. All these studies suggest that we should consider the interplay of these two roles when investigating the functionality of AMPK **(Figure 3)**.

Although in vitro and in vivo results are encouraging, whether the effects of AMPK activation have a similar therapeutic action in humans living in a thermoneutral environment is not clear. In addition, many mechanisms of AMPK in thermogenesis remain to be investigated, such as determining whether AMPK is involved in non-shivering thermogenesis independent of UCP1, including creatine and calcium round-trip. Moreover, clinical use of AMPK in the treatment of obesity is also confronted with many difficulties, such as how to perform site-specific targeting of AMPK in the human hypothalamus.

### mTOR signalling pathway

mTOR is involved in many important metabolic processes including lipogenesis and energy expenditure in BAT 51, 52. The most critical components of mTORC1 are the regulatory associated proteins of mTOR (Raptor) and the DEP domain-containing mTOR interacting protein (Deptor) and most upstream or downstream stimulation works through these two mTORC1 cores 53. mTORC2 shares Deptor with mTORC1 but has a unique element: Raptor-independent companion of mTOR (Rictor) 53. Raptor binding with the mTOR substrate motif is necessary for effective catalytic phosphorylation of mTOR. mTORC1/2 signalling regulates lipolysis, lipogenesis and mitochondrial function, and controls thermogenic gene expression to influence thermogenesis 54. Furthermore, mTOR also regulates autophagy 55.

The role of mTOR signalling in adipose thermogenesis is still unclear. Raptora^P2-Cre^ mice, a type of mice with adipocyte-specific deletion of Raptor, were found to show increased the expression of browning and thermogenic genes in WAT, and to have increased energy expenditure 56. In contrast, *Raptor^Adipoq-Cre^* mice, another type of mice with adipocyte-specific deletion of Raptor, also exhibit increased UCP1 expression and browning in WAT, but have no increase in energy expenditure 57, 58. The BAT mass and expression of thermogenic genes are decreased in *Raptor^Adipoq-Cre^* mice which suggests that mTORC1 is required for BAT formation and maintenance 57. All these studies indicate that the differential effects of mTORC1 on thermogenesis in WAT and BAT may be mediated via a noncell-autonomous mechanism. The underlying mechanism by which mTORC1 functions in WAT and BAT remains enigmatic and needs further study.

Therefore, it is not difficult to understand that mTORC1 can be activated by many regulators, such as growth factors, oxygen, amino acids and certain signalling pathways, such as WNT, Hippo and Notch. The most classic pathway by which mTORC1 participates in lipid metabolism is the binding of insulin-like growth factor and insulin to its receptor, which activates phosphatidylinositol-3-kinase (PI3K)/Akt signalling, inhibits Tsc 1/2, and leads to activation of GTP-RHEB, increasing the activity of mTORC1 against various substrates (including 4E-BP, ULK1, Lipin1, S6K and Grb10). Knockout of the Tsc 1 gene activates mTORC1 signalling to inhibit the expression of UCP1 and thermogenic genes in BAT 59, 60. Specific destruction of Grb10 expression in BAT enhances mTORC1 signalling, reduces the core body temperature and cold tolerance of mice, and weakens the expression of thermogenesis genes induced by cold in BAT 61. Similarly, white adipocytes of* S6K1^-/-^* mice also showed increased UCP1 expression 62. Recent studies have found that NP-cGMP signalling can also activate mTORC1 through PKG, thereby inducing adipose browning 63. Many recent studies have found that certain regulatory factors, including SNS, T3, and Mark4, can affect autophagy by regulating mTOR, ultimately regulating thermogenesis 64-66.

**Figure 4:**
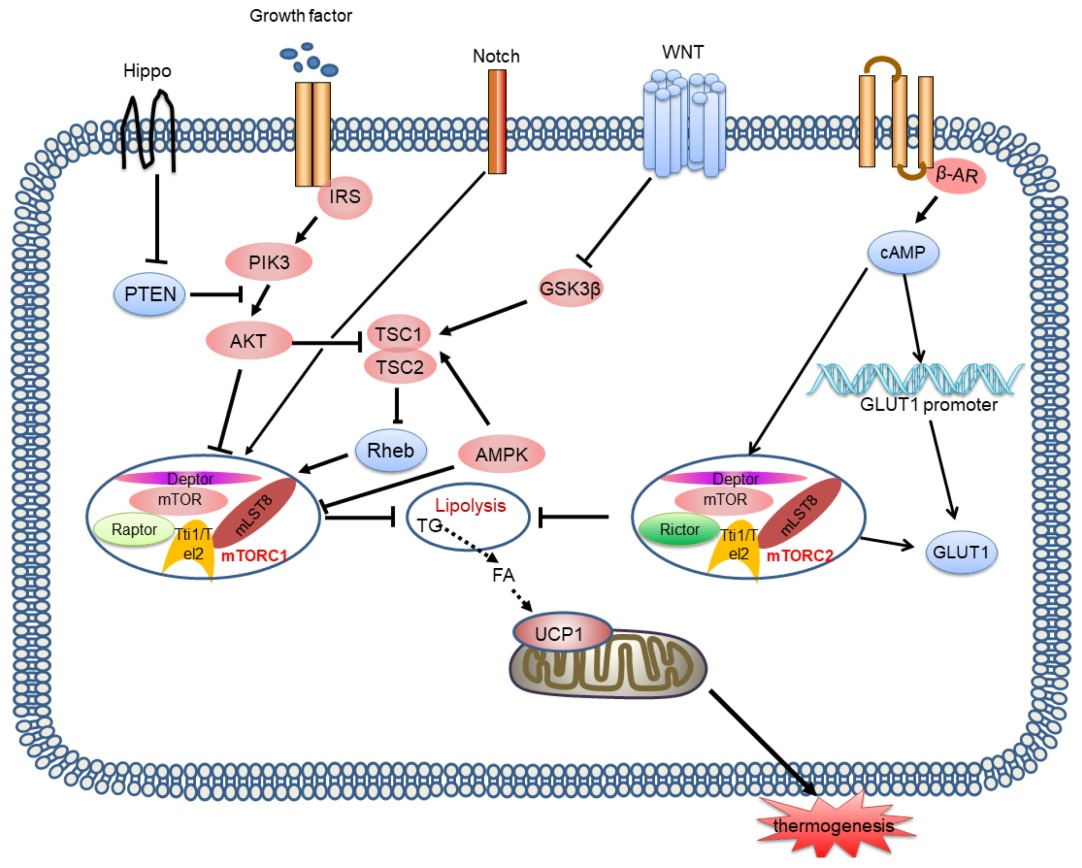
** mTORC1 and mTORC2 signalling pathways and their signalling networks.** The mTORC1 and mTORC2 signalling pathways are involved in thermogenesis by inhibiting lipolysis and regulating thermogenic gene expression. Growth factors activates the PI3K-AKT-TSC2-mTORC1 pathway. Wnt signalling inhibits the activation of GSK3β, which phosphorylates TSC2, resulting in mTORC1 stimulation. Notch signalling also affects mTOR activity. AMPK phosphorylates TSC2 resulting in inhibition of mTORC1 activity. Hippo activates mTORC1 signalling through PTEN suppression. mTORC2 is also involved in controlling glucose homeostasis. mTORC2 is stimulated by β-AR and then activates glucose metabolism and lipid oxidation, which is associated with thermogenesis. mTORC2 stimulates GLUT1 transport to the plasma membrane and increases glucose uptake. mTOR: mammalian target of rapamycin; PI3K: phosphatidylinositol-3-kinase; GSK3β: glycogen synthase kinase 3β; TSC2: tuberous sclerosis complex 2; PTEN: phosphatase and tensin homologue; GLUT1: glucose trans porter-1

In contrast to mTORC1, few studies have investigated the effect of the mTORC2 signalling pathway on BAT thermogenesis. Studies suggest that mTORC2 signalling is stimulated by β-AR or cold and then activates glucose metabolism and lipid oxidation in BAT, which is associated with thermogenesis 67. However, mTORC2 stimulates transport of glucose trans porter-1 (GLUT1) to the plasma membrane, increasing glucose uptake, irrespective of the classical insulin-PI3K-Akt pathway 68. Recently, Su, et al. showed that Rictor deletion in the BAT of mice inhibited lipid synthesis, and facilitated lipid catabolism and thermogenesis by activating the FoxO1 transcription factor, which is related to the mTORC2 substrate SGK, driving sirt6-mediated deacetylation of FoxO1 69. Similarly, we cannot determine whether mTORC2 interacts with mTORC1 to influence heat production by BAT. In the future, we must explore more direct and targeted models and methods. Moreover, the crosstalk between mTORC1 and mTORC2 in thermogenesis is not clear **(Figure 4)**.

### TGF-β/BMP signalling pathway

Transforming growth factor-β/bone morphogenic protein (TGF-β/BMP) signalling participates in most cellular processes and plays an important role in all metazoans. The TGF-β family consists of TGF-β, BMPs, and activin/inhibin and classical TGF-β/BMP signalling has a core that involves TGF-β/BMP ligands, signal transducers (Smads) and receptors (type I and II) 70.

Binding of Smad3 to PGC-1 inhibits its transcription, causing TGF-β/BMP signalling to inhibit brown adipocytes differentiation and thermogenesis. *Smad 3^-/-^* mice showed enhanced insulin sensitivity and reduced obesity, insulin resistance, and hepatic steatosis 71. The absence of TβRI promotes the formation of beige fat and reduces the harmful effects of HFD feeding 72. BMP4 promotes differentiation of human adipose stem cells into beige adipocytes, but decreases the expression of UCP1 and PGC-1α in BAT 73. Recently, BMP4 was found to have no effect on established obesity phenotypes, suggesting that BMP4 has a greater effect on brown adipocyte differentiation 74. BMP8B controls energy balance and is dependent on the degree of AMPK activation in the hypothalamus 75. In addition, other members of the BMP family are also involved in metabolism. For example, BMP7 and BMP8a can promote BAT thermogenesis, but the thermogenesis induced by BMP8a only appears in female mice, which may be because BMP8a-induced thermogenesis is mediated by oestrogen; hypothalamic BMP9 inhibits glucose production through a central pathway; Noggin, the extracellular inhibitor of BMP, was found to promote WAT browning and BAT thermogenesis 73, 76.

**Figure 5:**
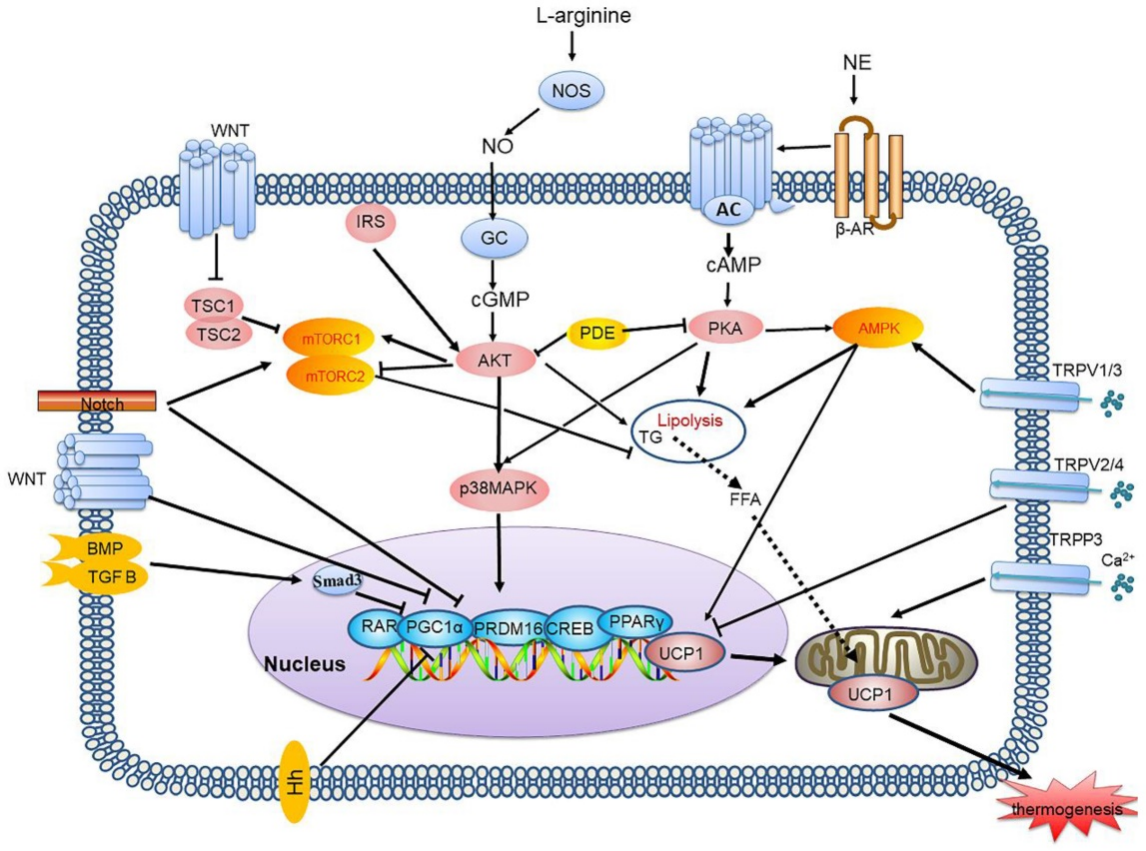
** Thermogenesis signalling pathways network.** Each signalling pathway plays an important role in adipocyte thermogenesis. Binding of Smad3 to PGC-1 inhibits its transcription, causing TGF-β/BMP signalling to inhibit brown adipocyte differentiation and thermogenesis. TRP channels transport Ca^2+^ and can be regarded as managers of intracellular Ca^2+^ levels, TRPV1/3 activate thermogenesis and TRPV2/4 inhibit thermogenesis. TRPP3 can enhance mitochondrial function. Notch signalling can regulate thermogenesis by influencing mTOR activity and directly inhibiting thermogenic gene expression. Hh inhibits WAT browning by downregulating thermogenic gene expression. The Wnt/β-catenin pathway inhibits adipogenesis and thermogenic programs by inhibiting transcription factors. PGC-1: PPARγ coactivator-1α; TGF-β/BMP: transforming growth factor-β/bone morphogenic protein; TRP: transient receptor potential; Hh: Hedgehog; WAT: white adipose tissue

How the balance of anti-adipogenic and pro-adipogenic TGFβ family proteins controls adipose progenitor differentiation by activating receptors and downstream factors, under different energy conditions is not clear. Further studies addressing the significance of TGFβ members in lipid biology and how their signalling components change would provide strong evidence supporting their potential role in obesity treatment **(Figure 5)**.

### TRP channels

TRP channels reside extensively on the membranes of various cells 77. TRP channels transport Ca^2+^ and can be regarded as managers of intracellular Ca^2+^ levels. Channels mediate Ca^2+^ influx in different ways, influencing several Ca^2+^ dependent signalling pathways and thereby affecting cell metabolism, differentiation, and gene expression 77, 78.

TRPV1, the first TPR channel to be identified in adipose tissue, is activated by medium temperature (≥43℃), and by various other stimuli such as low pH, capsaicin, and inflammatory factors 77. Capsaicin, a TRPV1 activator, promotes the expression of thermogenic genes in BAT by stimulating Ca^2+^ influx 79. However, studies have also found that TRPV1 in the nucleus of the solitary tract inhibits BAT thermogenesis in HFD rats 80. All these studies indicate that the effects of TRPV1 on obesity are complex. TRPV1 not only exists in adipose tissue, but also influences the CNS and gastrointestinal tract, which suggests that TRP channels in different tissues may have different effects and that the gut-brain axis may be an approach for obesity treatment.

TRPV3 expression was found to be significantly decreased in BAT and WAT, while TRPV2 and TRPV4 expression was increased, which shows that TRPV3 is similar to TRPV1 and that TRPV2/4 are opposite to TRPV1^81^.

TRPV2 is a nonselective calcium-permeable cation channel that is activated by toxins and high temperatures (above 52°C) 82. The expression of thermogenic genes was decreased in TRPV2 deficient mice and TRPV2 mRNA was detected in brown fat cells in vitro 83. However, studies also found that TRPV2 agonists inhibited the differentiation of brown adipocytes 84. This opposite effect on obesity may be related to Ca^2+^ influx: excess Ca^2+^ influx has a negative effect on BAT differentiation but is good for maintaining normal BAT thermogenesis.

The TRPM8 channel is a unique TRP channel that is induced by low temperature (below 26-28°C) stimulation 85. Menthol, a TRPM8 agonist, was found to activate TRPM8, resulting in PKA activation, UCP1 upregulation and increased thermogenesis 86. Meanwhile, inhibition of mitochondrial uncoupled respiration by streptomycin was achieved by inhibiting TRPM8-mediated calcium transport 87. In addition, TRPM8 was found to be involved in BAT clock regulation similar to TRPV1 and when TRPM8 was deficient, BAT clock regulation was disordered, resulting in a decrease in UCP1 expression 88.

Other TRP channels also play a role in thermogenesis **(Figure 5)**. TRPC1 is a possible target of PPARγ that promotes BAT thermogenesis 89. TRPP3 enhances mitochondrial function and promotes BAT differentiation 87. TRP channels respond to multiple environmental stimuli, such as temperature, food ingredients and poisoning, which indicates that the mechanism by which TRP channels are regulated is intricate. TRP channels have different effects in different tissues and even in different stages of adipose tissue differentiation, and elucidating the detailed mechanisms that regulate TRP channels might be difficult. Meanwhile, natural product ingredients regulate the function of TRP channels, and further investigation of the potential principle underlying their roles in obesity is urgent.

### Wnt/NF-κΒ/Notch/Hh signalling pathway

The Notch signalling pathway is a highly conserved pathway that is important for many cellular processes including survival, proliferation and differentiation 90. Previous studies have suggested that the Notch pathway inhibits the browning of WAT 90, 91. Bi et al. found that Notch signalling inhibits the transcription of thermogenic related genes, including Prdm16 and Ppargc1a in WAT 92. Huang et al. found that the reduction in subcutaneous adipose tissue expansion in pigs is mediated by inhibition of Notch signalling 93. However, a recent study suggested that Notch signalling promotes PKA activation and thermogenic gene expression in BAT, which is the opposite of the effects reported in previous studies in WAT 94. In addition, the researchers showed that Ras homolog enriched in the brain (Rheb) is a GTP-binding protein that promotes thermogenesis in BAT via activation of the Notch signalling pathway. How to explain the opposite mechanisms has not been determined, and further mechanisms have been lacking, including how Notch signalling regulates browning, and whether it controls adipocyte precursor differentiation or mature cell interconversion. We hypothesize that Notch signalling may be regulated by other regulators or pathways, or that there are distinct Notch receptors in WAT and BAT that have not yet been found. This should be explored in more depth. In short, Notch signalling plays a role in BAT thermogenesis or in the browning of WAT **(Figure 5)**.

Previous studies have shown that the Hh pathway is highly conserved in fat and is expressed in both fly and mouse fat. The Hh pathway blocks the early steps of adipogenesis, downregulates the adipogenic transcription factor PPARγ and induces the expression of osteogenic transcription factors 95. In this study, the researchers found that the Hh signalling pathway blocks differentiation of brown preadipocytes and promotes differentiation of preadipocytes towards skeletal muscle, thus inhibiting BAT formation in the body 96. Leptin also induces WAT browning by suppressing the Hh signalling pathway 97. However, in mature osteoblasts, upregulated Hh signalling was found to activate the endocrine action of bone-derived PTHrP, which causes continuous acceleration of bone remodelling and WAT browning for increased energy consumption 98. Although the effect is different, studies suggest that Hh signalling is involved in thermogenesis through WAT browning but not in the BAT thermogenic program** (Figure 5)**.

The classical Wnt/β-catenin pathway inhibits adipogenesis and thermogenic programs by inhibiting transcription factors including PPARγ, CEBPα and PGC1α 99-101. The Wnt/β-catenin pathway is a major axis, and many signalling pathways are connected through the Wnt/β-catenin pathway by regulating the expression of specific factors in the Wnt/β-catenin pathway. For example, lysine-specific demethylase 1 (LSD1) promotes brown fat formation via demethylation of H3K4 in the promoter region of the Wnt signalling module, thereby inhibiting the Wnt pathway 102. STAT3 induces differentiation of primary brown preadipocytes during the induction phase. Loss of STAT3 leads to upregulation of the Wnt ligand, and inhibition of the Wnt/β-catenin pathway can restore differentiation 103. Overall, the Wnt signalling pathway plays a vital role in thermogenesis and can be a therapeutic target for obesity and other associated metabolic complications **(Figure 5)**.

The NF-κB signalling pathway is responsible for induction of inflammatory genes and innate immunity, including a family of transcription factors 104. Zhang et al. found that dysregulation of NF-κB was mediated by SOCS3 in the hypothalamus 105, suggesting that NF-κB signalling is involved in energy balance. NF-κB signalling was shown to involve activation of oxidative phosphorylation by upregulating mitochondrial synthesis to distribute energy 106. Immediate early response gene X-1 (IEX-1) is a downstream target of NF-κB. IEX-1 inhibits WAT browning and activates thermogenic programs in WAT by promoting selective activation of fat macrophages 107. Homeobox a5 (Hoxa5), a developmental transcription factor, promotes WAT browning by inhibiting the tenascin C (TNC)-mediated Toll-like receptor (TLR) 4/NF-κB pathway and activating the BMP4/Smad1 pathway 108. Overall, the NF-κB signalling pathway may be a target for thermogenesis and associated metabolic diseases. This also suggests that the link between inflammation and thermogenesis is a worthwhile direction for obesity treatment **(Figure 5)**.

## Potential therapeutic applications in BAT

Adipose tissue is indispensable for total energy homeostasis, and adipose tissue dysfunction leads to metabolic diseases. Because of the ability of brown or beige adipocytes to expend energy, adipose tissue could be useful in treating obesity and other metabolic-related diseases. BAT and beige fat are involved in thermogenesis, and BAT and beige fat cells could potentially be activated as a therapeutic approach via several signalling pathways or certain regulatory factors. Nevertheless, recruitment and activation of human brown or beige adipocytes remain a challenge.

The classical method is cold exposure. Acute cold exposure (10°C, 4 h) induces UCP1-mediated thermogenesis-dependent glucose utilization by affecting amino acid metabolism in BAT 109. Chronic cold exposure (6°C, 10 days) has also been shown to activate glucose oxidation in BAT and WAT browning 110. Previous studies have found that intermittent cold exposure increases BAT thermogenesis 111. However, this time-consuming technique is uncomfortable and would be undesirable due to the increased cardiovascular risks of atherosclerotic plaque growth or instability 112. Moreover, further research is needed to determine whether these benefits will be sustained over the long term. After all, there are many ways in which the body can sense stimuli, control physiological responses, and ultimately adapt to the environment.

Pharmacotherapy to activate thermogenesis is an attractive choice. β3-AR agonists have been investigated for obesity treatment. The most common β3-AR agonists used in experiments to stimulate thermogenesis in brown adipocytes are CL316243, BRL-37344, and L-796568. Mirabegron, which is a β3-AR agonist applied for bladder hyperactivity therapy, was shown to activate BAT in rats and humans 113. However, Sui, et al. showed that the clinical dose of mirabegron induces BAT excitation and WAT browning and thereby leads to atherosclerotic plaque development 114. One study reported that RepSox, an inhibitor of TGFβ-RI, induces fat generation in mouse embryonic fibroblasts (MEFs) grown in fibroblast culture medium 115. Troglitazone, a PPARγ activator, promotes browning of WAT by activating TRPV1 and causing deacetylation of PPARγ 116. Rapamycin inhibits mTOR signalling. Recent studies have shown that short-term rapamycin treatment can lead to a variety of metabolic syndromes, such as hyperlipidaemia and insulin resistance, while prolonged treatment can lead to beneficial metabolic changes, including reduced obesity, increased insulin sensitivity, and improved blood lipids 117. These results suggest that the duration of rapamycin treatment might have different effects on metabolism, and that rapamycin has limitations in application for obesity disease. Nitrate is a substrate for NO production. Fatemeh, et al. showed that long-term nitrate administration has favourable effects on adiposity by increasing BAT and decreasing WAT in normal female rats 118. Some of these drugs have poor targeting, some have poor efficacy, and some have a variety of side effects. All these factors make them unsuitable for use in humans.

Currently, natural products targeting thermogenesis for treatment of obesity in the clinic have attracted public attention. How to influence thermogenesis through various signalling pathways is increasingly being studied.

In regard to the natural products that induce BAT thermogenesis, capsaicin must be the first compound people considered. Capsaicin, the most commonly occurring capsaicinoid, is a representative agonist of TRP 119. Classical research has demonstrated that oral administration of capsaicin activates TRP channels, especially TRPV1, in sensory neurons of the gastrointestinal tract, provokes thermogenesis via a β-AR-mediated pathway in BAT (TRP-SNS-UCP1 axis) and triggers browning of WAT 120, 121. Recent studies have also found that capsaicin can activate intracellular Ca^2+^ rise via TRPV1 channels, promote CaMKII/AMPK phosphorylation, and then activate SIRT-1, which facilitates interaction of PPARγ and PRDM-16 to enhance WAT browning and BAT thermogenesis 42, 79, 122. In addition to capsaicin, other natural product ingredients have agonistic activity towards TRPV1, including royal jelly (RJ) 123, 124, and sulphur-containing compounds in durian 125. In addition to TRPV1, they also activate TRPA1, which is a member of the TRP family. Cinnamaldehyde (CA) 126, menthol 127, and allyl isothiocyanate 128, among others, have agonistic activity towards TRPM8 and TRPA1, and thus may also have the potential to activate BAT thermogenesis. An introduction to these natural product ingredients can be found in **Table 1**, but will not be detailed here.

The AMPK signalling pathway is also involved in the BAT activation stimulated by many natural product ingredients, such as resveratrol, curcumin 134, EGCG 167 and berberine 154. Resveratrol is a phenylpropanoid present in various foods including red cabbage, spinach, berries, red wine, and peanuts. Previous studies have suggested that resveratrol contributes to anti-obesity effects by activating the AMPK-SIRT1-PGC-1α axis 171-174. Recent research has also found that resveratrol promotes UCP1 expression and browning in a p38 MAPK-dependent but SIRT1-independent manner 175. In addition, Hui, et al. reported that resveratrol partially enhances BAT activation and WAT browning via the gut microbiota-BA-TGR5/UCP1 pathway 176. Many other natural product ingredients also promote thermogenesis through the AMPK signalling pathway, and we summarize them in **Table 1.**

Natural product ingredients that promote thermogenesis are involved in multiple and overlapping signalling pathways. The current research only scratches the surface. Further research is needed to understand the anti-obesity mechanism of natural products in terms of thermogenesis. A growing number of natural products, such as curcumin 134, thymol 145, magnolol 144 and albiflorin 146, have been shown to improve obesity by affecting the thermogenesis of brown adipose tissue through a variety of signalling pathways, and we will not go into detail here. With the development of methods and technologies, the mysteries of thermogenic pathways and their integration and control have begun to become apparent. Understanding human obesity, improving the chances of finding effective treatments and greatly reducing safety risks is crucial. Therefore, future research on thermogenesis in BAT will further develop our understanding of BAT physiology and therapeutic potential.

The mechanisms underlying the anti-obesity action of natural products are as complex as the mechanisms underlying the thermogenesis signalling pathway. We have confidence in natural products, which likely offer safer and more effective ways to treat obesity based on thermogenesis in the future.

## Conclusion and prospects

Obesity is harmful to human health and leads to other diseases. Since the discovery of functional BAT in adults, targeting BAT has become a potential method to improve obesity and metabolic diseases. Further investigation of thermogenic signalling pathways is bound to apply to the treatment of obesity. Exploring the thermogenic signalling pathway is an important direction and approach to prevent obesity and related metabolism-related diseases.

However, further research is needed to clarify the importance and necessity of thermogenesis in humans and its potential applications in relation to the treatment of metabolism-related diseases. We have the following thoughts on future research on the signalling pathways related to thermogenesis:

1. Experiments studying the process by which certain pathways result in thermogenesis could be designed to discover regulators at committed steps of thermogenesis and BAT differentiation. Many signalling pathways, such as the mTOR pathway, have differential effects on thermogenesis which may be mediated via a noncell-autonomous mechanism and regulated by many upstream and downstream regulators. Therefore, it seems more reasonable to regulate the upstream and downstream regulators than to regulate the mTOR pathway directly.

2. The gut-brain-BAT axis may be an approach in the study of thermogenesis. Current understanding of the role of the gut-brain axis in energy balance regulation has received much attention and is a new direction in the treatment of obesity.

3. Study of the potential mechanism underlying inflammation in BAT thermogenesis is an important direction. Obesity is also a chronic systemic inflammation condition, and inflammatory pathways, such as the NF-κB pathway, are also involved in BAT thermogenesis and WAT browning. The interplay between inflammation and thermogenesis could lead to identification of novel signalling pathways.

4. The animal models and experimental systems used in the study of signalling pathways are the important basis. Different gene knockout mouse models may lead to radically different results, and the results from rodent studies are difficult to translate to human subjects. This requires discovery of other reliable animal models.

5. Research to reveal the specific mechanisms of age-related decreases in BAT could be a viable and effective way to recruit and activate human thermogenesis.

Nutraceutical targeting of BAT is by far the easiest and most likely effective method to treat obesity through thermogenesis. Application of natural medicines in obesity treatment is increasingly extensive, and research on the effect of natural products on BAT thermogenesis has gradually become a hot spot. Studying how natural ingredients affect thermogenic signalling pathways could lead to an understanding of the curative effects of natural ingredients on weight loss. However, it is undoubtedly complicated and difficult, and many questions remain.

1. Thermogenic signalling pathways interact with each other, and thus, the process is very complex. Moreover, the low specificity of drug targeting may lead to unexpected adverse reactions. Even the duration of action may lead to completely different outcomes. Much research is needed to explore the various signalling pathways and their interactions, and to better understand the mechanisms underlying the effects of natural products.

2. Currently, most natural products are available only as over-the-counter nutritional supplements, and there is a long way to go before they can be upgraded to pharmacological therapy.

3. Natural products are not sufficient as an acute, short-term, and safe medicine for obesity, and too many external factors affect long-term treatment with natural products. This is obviously unrealistic.

Therefore, much research is needed to explore the various signalling pathways involved in thermogenesis and their interactions, and to better understand the mechanisms underlying the effects of natural products.

## Figures and Tables

**Table 1:** Summary of natural products reported in recent years and the mechanisms of their active ingredients that promote non-shivering thermogenesis via signalling pathways^129^.

Signalling pathways	Source	Dietary component	Mechanism of action	Models	Ref
cAMP-PKA	Korean ginseng	Ginsenosides (GEF)	inhibit fat accumulation and increases energy expenditure in white adipocytes through PKA activation	Mouse 3T3-L1 pre-adipocytes, Mouse primary subcutaneous adipocytes (SAT)	130
cAMP-PKA	Bitter melon seed oil (BMSO)	Cis-9, trans-11, trans-13 isomer of conjugated linolenic acid	activate PKA and program cell death in WAT	Male C57BL/6JN mice (7 weeks old)	131
cAMP-PKA	Black mustard seeds, wine, and vinegar	Sinapic acid (SA)	stimulate mitochondrial biogenesis, WAT browning and lipolysis via the p38 MAPK-CREB pathway; activate thermogenesis via PKA-CREB signalling	3T3-L1 pre-adipocytes; BAT cells	132, 133
cAMP-PKA, AMPK	Turmeric	Curcumin	induce browning via an AMPK-mediated pathway; lead to lipid metabolism and energy homeostasis by increasing cAMP-PKA/CREB signalling pathways; promote adipogenic differentiation of preadipocytes and mitochondrial oxygen consumption	3T3-L1 preadipocytes, Male C57BL/6 J mice (6 weeks old), Primary adipocytes, Mouse brown adipocytes, Male* db/db* C57BL/6 mice	134-136, 137
PKA-p38MAPK-CREB, AMPK	*Mangifera indica*	Mangiferin (MF)	promote thermogenesis in brown preadipocytes via activation of AMPK and PKA-p38 MAPK-CREB Signalling pathways	C3H10T1/2 MSCs, Brown preadipocytes	138, 139
cAMP-PKA, AMPK	Fruits of hazel trees	Filbertone (C_8_H_14_O)	induce cAMP and then activate PKA, thus inducing phosphorylation of AMPK and CREB and thereby inhibiting adipogenesis, resulting in increased thermogenesis	Male C57BL/6N mice (5 weeks old), 3T3-L1 pre-adipocytes	140
PKA-p38 MAPK	Cinnamon	Cinnamaldehyde (CA)	activate PKA Signalling, increase expression of thermogenic genes and induce phosphorylation of HSL and PLIN1	Primary preadipocytes; Human adipose-derived stem cells (hASCs)	126
β3-AR-MAPK, AMPK	Many plants	Quercetin	promote UCP1 expression through the β3-AR and AMPK pathways	Male C57BL/6 mice (4 weeks old), Primary brown preadipocytes	141
PI3K-Akt	*Ishige okamurae*	Ishige okamurae extract (IOE)	activate the PI3K/Akt pathway and increase the expression of pro-thermogenic genes	Male *db/db* and lean* db/+* mice (5 weeks old)	142
β3-AR-PKA, AMPK	various plants of more than 20 species, including fennel, anise, and star anise, and has been used for culinary purposes for centuries	Trans-anethole (trans-1-methyoxy-4-propenyl-benzene)	induce browning through activation of the β3-AR and AMPK-SIRT1 pathways	3T3-L1 preadipocytes, Male C57BL/6 mice (5 weeks old)	143
PKA, AMPK	Magnolia officinalis (magnolia bark)	Magnolol	prevent oxidative stress and promote WAT browning by activating PPARγ-, pAMPK-, and PKA-mediated pathways	3T3-L1 preadipocytes	144
PKA, AMPK	Numerous aromatic plants such as thyme species	Thymol (5-methyl-2-isopropylphenol)	induce WAT browning by activating β3-AR-PKA-p38MAPK; alleviate lipogenesis by activating the AMPK pathway	3T3-L1 preadipocytes	145
AMPK, PI3K/AKT	Paeonia lactiflora	Albiflorin (AF)	induce brown adipogenesis by activating the AMPK and PI3K/AKT Signalling pathway	human adipose tissue-derived mesenchymal stem cells (HAMSCs), Male C57BL/6J mice (4 weeks old), Brown preadipocytes	146
PKA, AMPK-p38 MAPK	Various plants, such as dill, vanilla, violet flowers, and black pepper	Piperonal (C_8_H_6_O_3_)	increase PKA Signalling in WAT, regulate glucose uptake by inducing the lactate-AMPK-p38 MAPK pathway; cause mitochondrial respiration regulation via UCP1 induction	C57BL/6N mice, mouse embryo 3T3-L1 fibroblast cells, mouse C2C12 myoblasts, rat L6 myoblasts, 3T3-L1 preadipocytes	147, 148
AMPK	Panax notoginseng saponins (PNS)	Ginsenosides Rb1, Rd, Re, Rf and Rg1 and notoginsenoside R1	increase BAT thermogenesis and beige adipocyte reconstruction by activating the leptin-AMPK/STAT3 Signalling pathway	Male C57BL/6J mice (4 weeks old), *db/db* and *ob/ob* mice	149
AMPK	Flaxseed	Secoisolariciresinol diglucoside (SDG)	increase AMPK activation pathway and thus activate thermogenesis and stimulate mitochondrial biogenesis/activation/fission	Male *db/db* mice (5 weeks old), Male C57BL/6J mice (4 weeks old), Brown adipocytes, Beige adipocytes, 3T3-L1 adipocytes,	150
AMPK	Citrus aurantiumLinné (CA)	-	induce adipogenesis and thermogenesis through AMPK activation	Male C57BL/6J mice (4 weeks old), 3T3-L1 preadipocytes, Brown adipocytes	151
AMPK, NF-кB, MAPK	Fruits and vegetables	Apigenin (AP)	enhance thermogenesis and browning via AMPK activation; reduce adipose tissue metabolic inflammation (NF-кB, MAPK)	Male C57BL/6 mice (3 weeks old)	152
AMPK-PGC1ɑ	Ginger rhizomes (Zingiber officinale Rosco)	Ginger extract (GE)	stimulate browning via the SIRT1/AMPK/PGC-1α pathway	Male C57BL/6 J mice (5 weeks old)	153
AMPK	*Rhizoma Coptidis*	Berberine (BBR)	promote brown adipocyte differentiation and BAT thermogenesis through the AMPK-PRDM16 axis; stimulate UCP1 transcription through AMPK activation	Male C57BL/6J mice (6 weeks old), Adipose-specific *AMPKα1/α2 KO* mice (AKO), Male *db/db* mice, Brown preadipocytes, Primary stromal vascular (SVF) cells, C3H10T1/2 cells	154, 155
AMPK-PGC1ɑ	Soy	Genistein	increase UCP1 expression and that of some biomarkers of browning via AMPK pathway activation	Male C57BL/6 mice (8 weeks old), 3T3-L1 preadipocytes	156
AMPK	*Humulus japonicus*	Aqueous extract of Humulus japonicus (AH)	stimulate browning and β-oxidation and attenuate hydrogen peroxide-induced oxidative stress via AMPK and PPARδ-mediated pathways	3T3-L1 preadipocytes	157
AMPK	Outer bark of a variety of tree species	Betulinic acid (BA)	facilitate energy expenditure, lipid oxidation and thermogenesis by activating the AMPK pathway	3T3-L1 mouse embryo fibroblasts, Brown adipocytes, Male C57BL/6 mice (6 weeks old)	158
AMPK	Fungus	Cordycepin (Cpn)	activate UCP1 expression through AMPK activation	Male C57BL/6 mice, 3T3-L1 preadipocytes	159
AMPK	Platycodi Radix (root of Platycodon grandiflorum)	Platycodin D (PD)	decrease adipogenic markers including PPARγ and CEBPα via AMPK pathway activation and increase thermogenic factors such as UCP1 and PGC1α	Male* db/db* mice and age-matched WT mice (5 weeks old), 3T3-L1 mouse embryo fibroblasts, Brown adipocytes, hAMSCs	160
AMPK	*Humulus lupus*	Xanthohumol (XN)	activate AMPK resulting in beiging of 3T3-L1 adipocytes, enhance lipolysis and inhibit adipogenesis	3T3-L1 mouse embryo preadipocyte cell, Primary human subcutaneous preadipocytes	161
AMPK	Raspberry (RAS)	polyphenols	stimulate the expression of thermogenic genes and beige adipocyte formation through the AMPK pathway	Male *Rosa^Cre^/AMPKα1^flox/flox^*C57BL/6 mice, and age‐matched WT mice (2 months old)	162
AMPK	*Angelica sinensis*	Vanillic acid (VA)	inhibit adipogenic factors through the AMPK pathway and decrease lipid accumulation by suppressing adipogenic factors	Male C57BL/6J mice (4 weeks old), Male* db/db* mice and age‐matched WT heterozygous mice(5 weeks old), Brown preadipocytes, HepG2 cells, 3T3‐L1 cells, Primary brown adipocytes	163
AMPK	Many essential oils	Farnesol	induce mitochondrial/peroxisomal biogenesis and thermogenesis by enhancing the AMPK Signalling pathway in BAT	Male C57BL/6 J mice (4/7 weeks old), Brown preadipocytes	164
AMPK	Many types of plants, microalgae, and some bacteria	Gallic acid [3,4,5-trihydroxybenzoic acid (GA)]	elevate thermogenic gene expressions and activate the AMPK/Sirt1/PGC1α pathway in interscapular brown adipose tissue	HepG2 cells, Male C57BL/6 mice (10-12 weeks old)	165
AMPK	Rhubarb	Chrysophanic Acid	activate the AMPK pathway and then suppress adipogenesis and induce thermogenesis	Male C57BL/6J mice (4 weeks old), 3T3-L1 preadipocytes, Brown adipocytes	166
NF-κB, TLR-4, AMPK	Green tea	(-)-Epigallocatechin-3-gallate (EGCG)	raise mitochondrial biogenesis in BAT; inhibit the NF-κB and STAT3 pathways; increase the expression of TLR-4 by suppressing the expression of Elf-1; increase mtDNA replication and AMPK activation in BAT	Male C57BL/6J mice (4 weeks old), Male BALB/c mice (8 weeks old), Peritoneal macrophages	167-169
AMPK	Many edible and medicinal plants such as pepper, celery, thyme, peppermint and honeysuckle	Luteolin	elevate thermogenic gene expressions and activate AMPK/PGC1α signalling in differentiated primary brown and subcutaneous adipocytes	Male C57BL/6 mice (4 weeks old), Primary brown adipocytes, Subcutaneous adipocytes	170
TRPA1, TRPV1	Durian	Sulphur-containing compounds (DEDS, DPDS, DETS DPTS, and PT)	induce Ca^2+^ responses in TRPA1- or TRPV1-expressing cells and then activate both TRPA1 and TRPV1	Human TRPA1- or TRPV1-expressing HEK cells	125
TRP	Royal Jelly (RJ)	10-hydroxy-Trans- 2-decenoic acid (HDEA) and Hydroxydecanoic acid (HDAA)	enhance thermogenic gene expressions; activate the TRP channels, specially TRPA1 in sensory neurons of the gastrointestinal tract; promote thermogenesis via β-AR-mediated pathway in brown and white adipocytes (TRP-SNS-UCP1 axis)	Male Wistar rats (3 weeks old)	123, 124
					

## Abbreviations

AMPK: AMP-activated protein kinase
ATF-2: activatingtranscriptionfactor-2
ATGL: adipose triglyceride lipase
AKT/cGMP: cyclic guanosine monophosphate
ANP: atrial natriuretic peptide
BNP: brain natriuretic peptide
BAT: brown adipose tissue
β-AR: β-adrenoceptor
CNP: C-type natriuretic peptide
cAMP: cyclic adenosine monophosphate
CEBPs: element-binding proteins
CNS: central nervous system
CPT1: carnitine palmitoyl transferase I
CD-NP: optimized designer natriuretic peptide
CREB: cAMP-response element binding protein
Dio2: Type II iodothyronine deiodinase
Dlk1: delta like non-canonical Notch ligand 1
eNOS: endothelial NO synthase
Elf-1: E74-like ETS transcription factor 1
FGF21: fibroblast growth factor 21
FA: fatty acid
Fox: Forkhead box
GLP-1: glucagon-like peptide 1
GC: guanylyl cyclases
GLUT: glucose transporter
HFD: high fat diet
HSL: hormone-sensitive triglyceride lipase
Hh: Hedgehog
HDAC3: histone deacetylase 3
IRE-1: inositol-requiring enzyme-1α
IRS: insulin receptor substrate
LSD1: lysine-specific demethylase 1
MCU: mitochondrial calcium uniporter
mTOR: mammalian target of rapamycin
NE: norepinephrine
NO: Noitric oxide
NP: natriuretic peptide
NPR: natriuretic peptide receptor
NF-κΒ: nuclear factor-κ-light-chain-enhancer of activated B cells
PDE: phosphodiesterase
PKA: protein kinase A
PLIN: Perilipin
PPARγ: peroxisome proliferator-activated receptor γ
PGC1α: PPARγ coactivator-1αl
PRDM16: PR domain zinc finger protein
p38 MAPK: p38 mitogen-activated protein kinase
sGC: Soluble GC
Smad3^-/-^: Smad3 gene knockout
S6K: S6 kinase
SNS: sympathetic nervous system
SIRT: silent mating type information regulator 2 homolog
Tsc1/2: tuberous sclerosis complex1/2
TGF-β/BMP: transforming growth factor-β/bone morphogenic protein
TRP: transient receptor potential
TBX1: T-box transcription factor
UCP1: uncoupling protein 1
WAT: white adipose tissue
XBP-1: X-box binding protein-1
